# Rehabilitation in a war zone

**DOI:** 10.2471/BLT.22.021122

**Published:** 2022-11-01

## Abstract

Supported by international partners, Ukraine is building on rehabilitation work initiated by the government prior to the current conflict. Fid Thompson reports.

Yurii Bondarenko (name changed at his request) became a casualty of the war in Ukraine on 2 September 2022. “I was at the market and suddenly there was this loud explosion,” says the 76-year-old geologist who was until recently a resident of the city of Bakhmut in the Donetsk region.

Yurii was thrown to the ground, smashing his right elbow. He managed to get back on his feet and make his way home, but then he passed out on the kitchen floor. “I was lying there for three days,” he says. “My son found me when he came back from a business trip.”

When Yurii regained consciousness, he found that he could no longer walk unaided.

Yurii is one of thousands of civilians to have sustained injuries in Ukraine since the beginning of the Russian Federation invasion on 24 February 2022. According to the Office of the United Nations High Commissioner for Human Rights (OHCHR), as of 2 October, at least 9132 civilians had been injured and 6114 killed (the OHCHR believes the real toll to be significantly higher).

Most of the casualties have resulted from shelling, missile strikes and aerial bombardment and many of the people injured have required rehabilitation supported by assistive technologies (AT).

Ranging from physical devices such as wheelchairs, prostheses, hearing aids and spectacles to digital products such as software and apps that facilitate communication, AT products are as diverse as the needs of the people who require them. In conflict zones, however, the drivers of demand tend to fall into three main categories.

“In Ukraine, the bulk of the demand for AT is driven by war-related trauma, the needs arising as people with disabilities flee danger,” explains Tetiana Lomakina, Presidential Adviser on barrier-free society issues, a position established in 2021 as part of a national “Without Barriers” initiative to include and empower people living with disabilities which was launched by First Lady Olena Zelenska.

“People are also having to cope with dramatic changes in their living conditions that include the loss of heating, water and electricity, and in many cases the complete destruction of their home,” Lomakina adds.

On the trauma side, the rehabilitation needs tend to be urgent and complex. “What we see most commonly are extensive burns and complex fractures caused by explosives,” says Pauline Falipou, an emergency rehabilitation specialist with Humanity & Inclusion (formerly Handicap International), an international nongovernmental organization (NGO) working in Ukraine. “The injuries sustained generally require immediate surgical intervention followed by intensive rehabilitation that has to start as soon as possible and should be followed for months if not years,” she explains.

“Rehabilitation was included in the response to the war from day one.”

Volodymyr Golyk

Since March 2022, Humanity & Inclusion has been supporting the rehabilitation and AT needs of injured people in Ukraine’s frontline hospitals, but the NGO also works to address the needs of people living with disabilities in the many communities affected by war, including those living in homes for the elderly and children.

The United Nations Children’s Fund (UNICEF) is also heavily committed in Ukraine, monitoring the well-being and safety of some 40 000 children – including children with disabilities – in institutions that have often found themselves caught up in the hostilities, necessitating rapid relocation. 

“Many of the children are in institutions because of a lack of family support services and inclusive education. Poverty is also a contributing factor,” says Fernando Botelho,  Assistive Technology Program Specialist at UNICEF’s New York headquarters. “So, evacuating and relocating them while ensuring that they can still access education, family support, health and other social services has often been extremely challenging.”

The World Health Organization’s engagement in Ukraine includes coordinating the rehabilitation response effort, collaborating with partners to provide on-the-ground training in early (i.e. post-intervention) rehabilitation, and working with the government and partners to establish a national rehabilitation centre for spinal cord injuries.

With funding from ATscale, a global, cross-sector partnership launched in 2018 to address gaps in access to AT, WHO has also developed AT kits based on assessments of the rehabilitation needs in the country. The first kit, AT6, comprises six items associated with trauma care – wheelchairs, crutches, walking frames, mobile toilets and shower chairs. Fifty of the kits were delivered with external fixators (medical devices used to consolidate fractures) to 11 hospitals in Eastern Ukraine in June, enough to support 2500 patients.

“The idea is to encourage the integration of rehabilitation services into trauma care,” explains Peter Skelton, Rehabilitation in Emergencies Focal Point at WHO’s Geneva headquarters. “When we send external fixators and bone saws to a trauma hospital, we’re also sending crutches and wheelchairs. So, you’re not just saving or amputating the limb, you’re also thinking about how the person is going to function and access essential services afterwards.”

A second-generation kit, AT10, includes items designed to meet the needs of internally displaced people, including static toilets, absorbent products, and three different types of catheter kits. As of 10 October, five such kits had been delivered to hospitals in Eastern Ukraine. UNICEF has developed similar AT packages for children.

The delivery of kits is not in itself enough to ensure the delivery of rehabilitation services. As Volodymyr Golyk, technical officer for disability and rehabilitation in WHO’s Ukraine office, puts it: “You can’t just give out crutches or wheelchairs. Properly trained health workers are required to make sure it is put to optimal use as early as possible, starting from the acute rehabilitation phase.”

To support health workers engaged in the rehabilitation response, the kits provided by WHO include instructions on how to prescribe, fit and train the recipient of the AT devices. Health workers can also access WHO’s online AT training platform and avail themselves of communication boards that are printed in Ukrainian. As mentioned above, health workers are also receiving on-the-ground training in early rehabilitation.

Efforts to boost the rehabilitation capacity of the Ukrainian health system are being greatly facilitated by the system itself, as Golyk is quick to point out. “The emergency rehabilitation response is building on what the Ukrainian government was already developing around assistive technologies and rehabilitation,” he says, noting initiatives that date back to the Russian Federation’s invasion of Crimea and include the training of rehabilitation professionals. “This has meant that rehabilitation was included in the response to the war from day one,” he adds.

“Early and comprehensive rehabilitation determines how quickly a patient can return to active life.”

Tetiana Lomakina

Capacity-building efforts initially focused on the needs of injured military personnel but have since been expanded to boost access for civilians.

This drive to increase access reflects a fundamental rethinking of emergency response strategy which emphasizes rapid response and comprehensive support. “The Ukrainian rehabilitation system was formed in a different era and under different conditions,” says Lomakina. “We know now that early and comprehensive rehabilitation determines how quickly a patient can return to active life, become independent, learn, work, and communicate.”

It is with a view to providing such care that the President’s office, working in collaboration with the Ministry of Health and the Ministry of Social Policy, is working on setting up inpatient rehabilitation departments with “AT cabinets” (AT units) in 24 hospitals across the country.

The units are to be staffed with multidisciplinary teams providing rehabilitation as well as guidance on the use of AT devices, and maintenance and follow-up services. “We now face the task in the middle of war to get the departments set up, train multidisciplinary rehabilitation teams, update legislation and fundamentally change the approach to rehabilitation,” Lomakina says.

Lomakina believes that the decentralization of rehabilitation service delivery could be of enormous benefit not only to those injured during the war, but also to the broader population requiring rehabilitation services. To that extent, it is closely aligned with the barrier-free society agenda that was being pursued by the First Lady prior to the invasion.

Much of the work required to achieve that agenda has been put on hold because of the war. Notable initiatives include the launch of a Large Construction National Program which emphasizes inclusive infrastructure facilities and public spaces such as a swimming pool accessible to people with disabilities that was built in Zolotonosha and a sports and rehabilitation centre that was being built in Krasyliv.

Lomakina is looking forward to getting these and other projects up and running again.

For Yurii Bondarenko, walking will be enough. With the support of the Humanity & Inclusion rehabilitation team located in Dnipro, Yurii began his journey back to full autonomy using a balance and mobility trainer. A physiotherapist taught him how to do balancing exercises and he was given an AT device in the form of a walking stick to help him on his way.

He and his son have decided to move to the Kyiv region “I have always been very active and getting back on my feet has really raised my morale,” Yurii says. “I am looking forward to going to the market again.”

**Figure:**
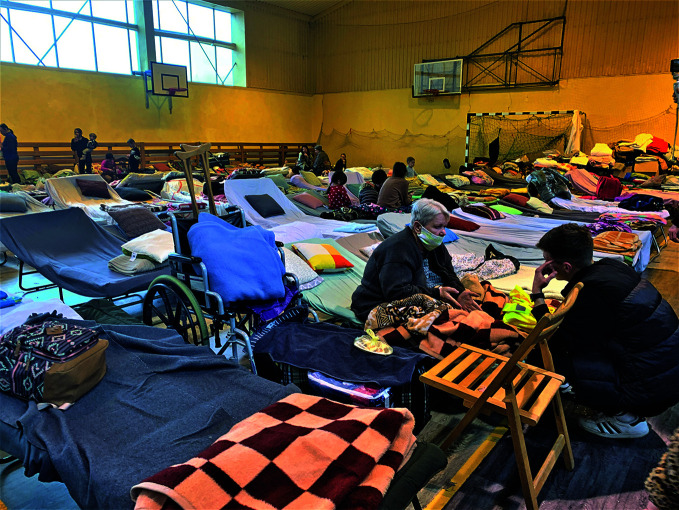
Ukrainian refugees at a reception centre in the village of Horodło, Poland.

WHO/Sarah Tyler

**Figure:**
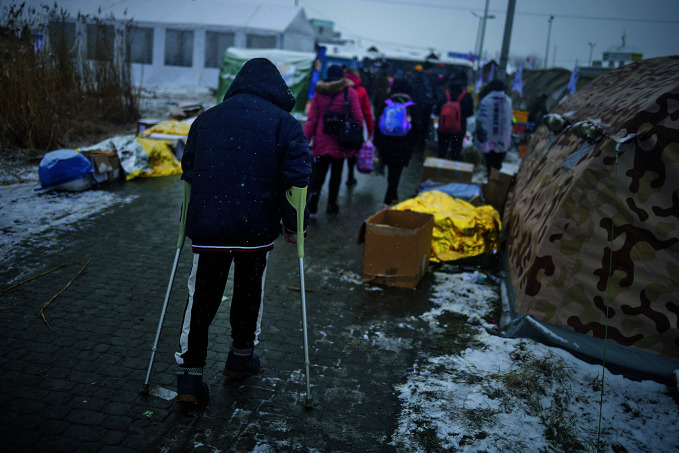
A man with crutches traverses the Medyka crossing between Ukraine and Poland.

Humanity & Inclusion/Tom Nicholson

